# Efficient Real-Time Isotope Identification on SoC FPGA

**DOI:** 10.3390/s25123758

**Published:** 2025-06-16

**Authors:** Katherine Guerrero-Morejón, José María Hinojo-Montero, Jorge Jiménez-Sánchez, Cristian Rocha-Jácome, Ramón González-Carvajal, Fernando Muñoz-Chavero

**Affiliations:** 1 Department of Electronic Engineering, University of Sevilla, 41092 Sevilla, Spain; kguerrero@us.es (K.G.-M.); jjsanchez@us.es (J.J.-S.); crirocjac@alum.us.es (C.R.-J.); carvajal@us.es (R.G.-C.); 2 Department of Electronic Engineering Computer Systems and Automatics, University of Huelva, 21007 Huelva, Spain; josemaria.hinojo@diesia.uhu.es

**Keywords:** nuclear spectroscopy, isotope classification, principal component analysis, random forest, real-time processing, SoC FPGA

## Abstract

Efficient real-time isotope identification is a critical challenge in nuclear spectroscopy, with important applications such as radiation monitoring, nuclear waste management, and medical imaging. This work presents a novel approach for isotope classification using a System-on-Chip FPGA, integrating hardware-accelerated principal component analysis (PCA) for feature extraction and a software-based random forest classifier. The system leverages the FPGA’s parallel processing capabilities to implement PCA, reducing the dimensionality of digitized nuclear signals and optimizing computational efficiency. A key feature of the design is its ability to perform real-time classification without storing ADC samples, directly processing nuclear pulse data as it is acquired. The extracted features are classified by a random forest model running on the embedded microprocessor. PCA quantization is applied to minimize power consumption and resource usage without compromising accuracy. The experimental validation was conducted using datasets from high-resolution pulse-shape digitization, including closely matched isotope pairs (^12^C/^13^C, ^36^Ar/^40^Ar, and ^80^Kr/^84^Kr). The results demonstrate that the proposed SoC FPGA system significantly outperforms conventional software-only implementations, reducing latency while maintaining classification accuracy above 98%. This study provides a scalable, precise, and energy-efficient solution for real-time isotope identification.

## 1. Introduction

The classification of isotopes with similar energy levels represents a major challenge in nuclear physics and materials science, especially for areas such as radiation monitoring, nuclear waste management, and medical imaging. Many applications in these areas demand rapid decision-making, where even minimal delays can jeopardize safety, disrupt operations, or reduce diagnostic effectiveness. Therefore, low-latency identification becomes a fundamental requirement. However, the spectral overlap between isotopes with similar energy emissions complicates classification, necessitating advanced signal processing and pattern recognition techniques [1,2]. To address these constraints, previous studies have emphasised the importance of real-time processing architectures. For instance, in radiation safety and security contexts, prompt identification of hazardous isotopes is crucial for triggering timely alarms and initiating mitigation actions [3]. Similarly, in beam experiments, where test campaigns requiring high data rates are conducted, it is important to maintain the detector’s acquisition speed to avoid data loss and ensure continuous operation [4]. Another relevant example is the FAZIA collaboration, which makes use of high-performance electronics to process the signals generated by the collision of radioactive ion beams in real time before they decay [5]. These examples demonstrate the need to develop high-performance hardware solutions that enable new low-latency processing strategies to be implemented directly on the device. Thus, real-time and hardware-accelerated classification systems are not only advantageous but essential in many cases to meet the operational demands of modern nuclear instrumentation.

System-on-Chip Field-Programmable Gate Arrays (SoC FPGAs) have emerged as a powerful solution for real-time and high-speed data processing by combining the parallel processing capabilities of FPGA logic fabric with the flexibility of an integrated microprocessor system [6]. These characteristics make them well-suited for handling large-scale spectral data in nuclear spectroscopy. In this work, the FPGA logic fabric is used to implement principal component analysis (PCA) [7] for feature extraction, reducing data dimensionality while optimizing both memory usage and computational efficiency. To further enhance system performance, quantization techniques have been applied to reduce power consumption, increase processing speed, and minimize resource utilization while ensuring that no loss of precision occurs.

Once the data have been reduced in dimensionality, they are transferred to the microprocessor embedded within the SoC FPGA. Then, a user-level application developed in Python 3 runs a random forest classifier using standard Python libraries [8]. This hybrid architecture efficiently distributes computational tasks, ensuring fast execution while maintaining classification accuracy.

The use of FPGAs and artificial intelligence methods for digital pulse-shape analysis in isotope identification has been previously explored. The authors in [2] proposed a classification method to discriminate between ^12^C and ^13^C using fully connected neural networks, providing an FPGA implementation. Later, ref. [1] extended this approach to classify ^12^C vs. ^13^C, ^36^Ar vs. ^40^Ar, and ^80^Kr vs. ^84^Kr using fully connected networks. While neural networks demonstrated high classification accuracy, they demanded significant FPGA hardware resources and introduced considerable latency, often spanning thousands of clock cycles. In a previous study [9], which serves as the basis for this work, PCA was proposed for dimensionality reduction before applying a classification method based on Support Vector Machines (SVMs).

It is important to note at this point that a direct comparison of FPGA resource utilization between a hybrid solution (hardware for PCA and software for random forest) and neural networks based entirely on hardware is difficult as both logic resources and the use of the integrated processor must be considered. However, thanks to the low hardware resource consumption of the PCA implementation, the proposed system can distinguish a greater number of isotopes due to the hybrid scheme since the random forest algorithm can be executed on another device as it is software-based.

Building upon that foundation, this study presents several key advancements over previous work:

- A hardware implementation of PCA has been developed, with a detailed study on quantization techniques to optimize power consumption and latency.
- The system is deployed on an SoC FPGA, leveraging its programmable logic for PCA and its microcontroller for classification.
- Random forest replaces SVM as the classification algorithm, proving to be more robust to aggressive PCA quantization.
- The complete system has been implemented on a real Xilinx FPGA, demonstrating reductions in complexity and latency by several orders of magnitude compared to neural-network-based approaches while maintaining classification accuracy.

This paper is structured as follows. Section 1 contextualizes the problem and its relevance. Then, Section 2 details the data collection and isotope measurement process. After describing the data collection, the methodology followed is presented in Section 3. Section 4 and Section 5 cover the definition and implementation of the proposed classifier based on PCA and random forest in an SoC FPGA, respectively. Finally, the conclusions are presented in Section 7.

## 2. Dataset and Isotope Measurement

This section describes the origin of the dataset used in this study and presents the problem of digital pulse-shape analysis (DPSA) for isotope discrimination. We detail the experimental procedures and equipment involved in data collection, providing insights into the nature and quality of the measurements.

The dataset was collected at the Grand Accélérateur National d’Ions Lourds (GANIL) using the CIME cyclotron to accelerate ions. A silicon detector, doped via neutron transmutation doping (NTD), captured electric current signals directly from the ion beam, eliminating the need for a target. The detector, 300 μm thick with a $200\;{\mathrm{mm}}^{2}$ active area, used a reverse configuration to improve temporal resolution [10]. Operating at 190 V with a depletion voltage of 140 V, it was connected to a low-gain preamplifier within a 300 MHz bandwidth. The output signals, recorded with the detector positioned 4 cm from the preamplifier, were digitized using an 8-bit ACQIRIS system capable of operating at sampling frequencies of up to 2 GHz. Data acquisition was performed at a sampling frequency of 1 GHz. The ACQIRIS system digitized and stored signals on a consistent amplitude scale, enabling easy comparison between ion pulses. Energy measurement was carried out using a peak-detection analog-to-digital converter (ADC), connecting the preamplifier’s charge output to standard analog shaping electronics.

Mixed-isotope beams with constant energy per nucleon were used. Isotope identities and mass numbers were determined through precise total energy measurements. A more comprehensive description of the experiment can be found in [11].

**Figure 1:**
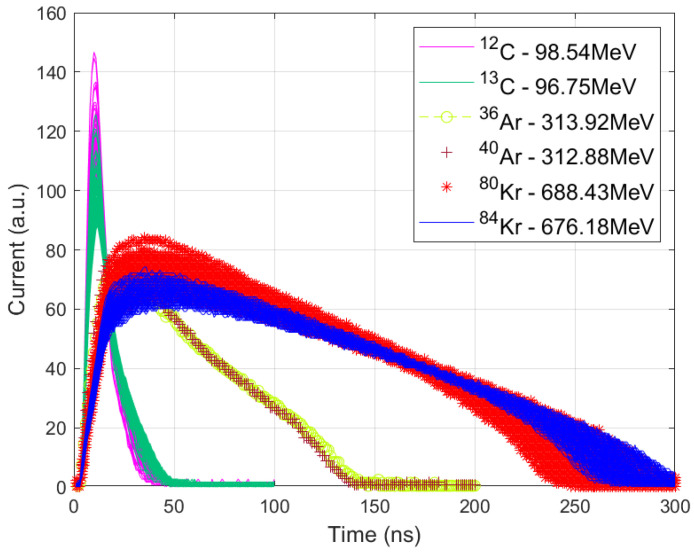
Current pulse waveforms for the three isotope pairs: ^12,13^C, ^36,40^Ar, and ^80,84^Kr.

Three isotope pairs with closely matched total energies posed significant classification challenges, as shown in Figure 1:

- ^12^C with an energy of 98.54 MeV versus ^13^C with an energy of 96.75 MeV.
- ^36^Ar (313.92 MeV) vs. ^40^Ar (312.88 MeV).
- ^80^Kr (688.43 MeV) vs. ^84^Kr (676.18 MeV).

The input features used for classification consist of digitized samples from the 8-bit ADC, as indicated in Figure 1. Specifically, 100 samples are used for the ^12^C/^13^C pair, 200 samples for ^36^Ar/^40^Ar, and 300 samples for ^80^Kr/^84^Kr. These samples represent the electric current pulse shapes, which contain critical information for isotope discrimination. It is important to note that the isotope pair ^36^Ar and ^40^Ar was particularly difficult to discriminate due to significant pulse-shape overlapping.

This paper presents a real-time method for discriminating between pairs of isotopes with similar energy, designed for minimal resource usage on a Xilinx Series 7 SoC FPGA. The approach utilizes the logic fabric to efficiently implement PCA for feature reduction and the embedded microprocessor to run the classification algorithm. This dual-layered approach, which combines hardware-accelerated PCA with lightweight software classification, enables real-time processing while maintaining low computational resource consumption, making it ideal for real-time applications.

## 3. Methodology

**Figure 2:**
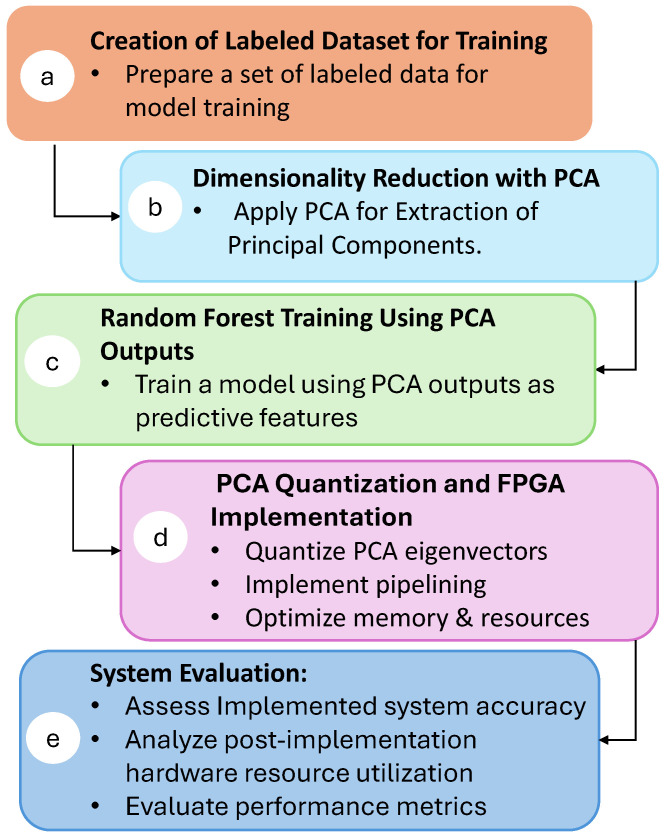
Schematic overview of the research methodology.

This section describes the process used to develop and implement the real-time isotope classification system. Figure 2 presents an overview of the workflow, from the initial preparation of the data to the performance evaluation of the FPGA-implemented system:

a. **Creation of Labeled Dataset for Training:** The process begins by forming a labeled dataset. Each entry includes the isotope label, name, and essential attributes, such as the numbers of protons (Z) and neutrons (N). The initial features consist of raw current pulse samples: 100 samples for ^12,13^C, 200 samples for ^36,40^Ar, and 300 samples for ^80,84^Kr.
b. **Dimensionality Reduction with PCA:** To achieve feature reduction, principal component analysis is applied to the dataset. PCA was chosen due to its high level of parallelism, which allows for an efficient implementation on hardware. By distributing the computational workload across multiple processing elements, PCA can be executed rapidly and with minimal latency, making it ideal for real-time applications. The details of this procedure are provided in Section 4.2.
c. **Random Forest Training Using PCA Outputs:** The output from PCA serves as input features for offline training of a random forest classifier. This machine learning algorithm is chosen for its robustness and ability to handle complex datasets. A complete explanation of this training process is provided in Section 4.3.
d. **PCA Quantization and FPGA Implementation:** To optimize the hardware implementation, the PCA eigenvector values are quantized, reducing resource usage without sacrificing classification accuracy. PCA calculation is implemented on the FPGA, taking advantage of pipelining techniques and memory optimizations to minimize latency and resources. The details of the quantization process and FPGA implementation are discussed in Section 4.4 and Section 5, respectively.
e. **System Evaluation:** The final step involves evaluating the implemented system. This includes assessing classification accuracy and analyzing the hardware resources used post-implementation. Key performance metrics, such as latency, resource utilization, and overall accuracy, are presented in Section 6. Additionally, a comparative analysis is presented regarding similar real-time FPGA implementations, demonstrating the advantages of the proposed architecture in terms of both efficiency and accuracy.

## 4. Random Forest Classifier Using PCA Outputs

Random forest was selected for this study due to its strong balance between classification accuracy, computational efficiency, and ease of implementation within the embedded microprocessor of the chosen SoC FPGA. Furthermore, its low computational complexity allows for efficient software implementation well-suited for real-time classification on embedded systems. This approach allows the system to identify the isotope type directly on the FPGA platform without requiring external data processing, enabling fully self-contained operation. The classifier’s robustness, even under aggressive dimensionality reduction via PCA, further supports its suitability for resource-constrained real-time applications.

### 4.1. Feature Reduction with PCA

**Figure 3:**
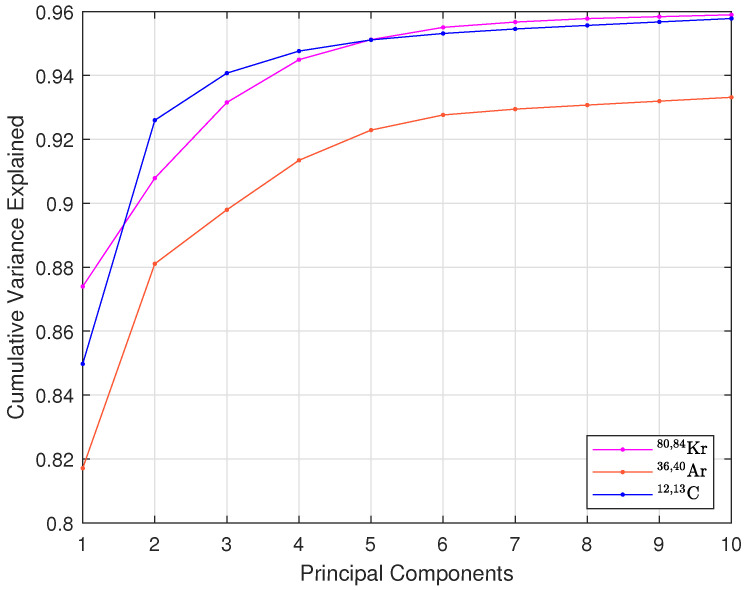
Cumulative explained variance of principal components for ^80,84^Kr, ^36,40^Ar, and ^12,13^C isotopes.

The first stage of the classification process applies PCA to the raw data to reduce its dimensionality while preserving the most relevant information. PCA is a well-established statistical technique that transforms high-dimensional datasets into a lower-dimensional space, capturing the underlying structure through a set of orthogonal principal components. These components retain the most significant variance in the data, enabling more efficient processing without compromising essential information. The dataset includes current pulse samples captured by an 8-bit ACQIRIS digitizer with a sampling interval of 1 ns. Table 1 summarizes the number of observations and features per isotope pair:

As is known, PCA involves computing the covariance matrix, extracting eigenvalues and eigenvectors, and projecting the data onto the principal components with the highest variance [12]. A detailed description is available in [9,13]. Dimensionality reduction in PCA involves selecting how many principal components (projections of the data onto the eigenvector directions) will be used as input features for the chosen classifier. To determine the optimal number of principal components, the cumulative explained variance was first analyzed, as shown in Figure 3. For most isotopes, over 95% of the variance is captured with the first six components. However, reducing this to four still preserves most of the critical information while significantly lowering computational demands. Cumulative explained variance shows how much information is retained or lost when excluding a principal component but does not indicate if the excluded data is correlated with the dataset labels. Therefore, variance analysis is used as a preliminary step, with final component selection guided by a loss function that balances classification accuracy and hardware efficiency.

Moreover, reducing the number of principal components has a notable impact on hardware efficiency. The lower the number of principal components, the lower the number of multiplications and additions that need to be carried out. It also simplifies the random forest classifier by reducing the number of input features, leading to lower resource usage. Therefore, the final selection of the number of principal components is made in Section 4.2, where PCA is combined with the random forest classifier. A loss function, incorporating classification accuracy, is used to balance model performance and hardware efficiency. This approach ensures an optimal configuration, validated using two key metrics: classification accuracy and a figure of merit (FOM), which will be described in next section.

### 4.2. Selection of Number of Principal Components

Before fine-tuning the random forest classifier, the second step focuses on selecting the optimal number of principal components (PCs) for each isotope pair. The target accuracy for this step is to match or exceed the performance reported in [1], where a fully connected neural network was used to classify the same dataset.

An extensive evaluation was conducted using random forest classifiers with default hyperparameters. Models were trained and tested with varying numbers of principal components (from one to ten) to observe the impact on classification accuracy. This assessment helps to identify the ideal trade-off between classification performance and hardware efficiency for FPGA integration.

**Figure 4:**
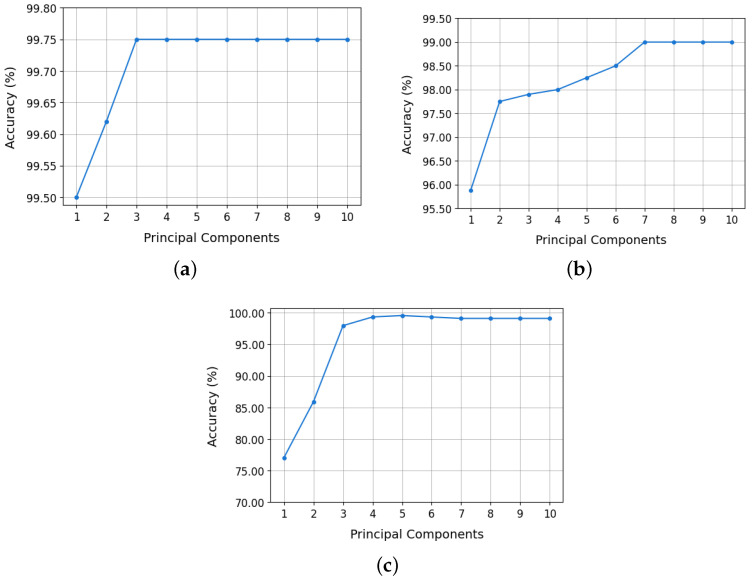
Random forest model accuracy as a function of the number of principal components: (**a**) ^12,13^C, (**b**) ^36,40^Ar, and (**c**) ^80,84^Kr.

The dataset was split 80/20 for training and testing, respectively, to ensure unbiased evaluation and prevent overfitting. Accuracy was used as the primary performance metric for all configurations. Figure 4 illustrates classification accuracy for each isotope pair across varying PCA configurations:

- **^12,13^C:** model accuracy stabilized at 99.75% using only two principal components. This value remained constant up to 10 PCs. Even with a single PC, 99.50% was achieved. These results are shown in Figure 4a.
- **^36,40^Ar:** accuracy improved significantly up to 7 PCs, plateauing at 99.00% from 7 to 10 PCs (Figure 4b). A reduced configuration with 2 PCs still achieved 97.75%, making it a practical choice for implementation.
- **^80,84^Kr:** accuracy increased progressively with the number of principal components, reaching a maximum value of 99.09% when using all ten PCs (Figure 4c). However, with only three PCs, an accuracy of 97.95% was achieved, which represents an adequate compromise between classifier performance and efficiency in the use of hardware resources.

Based on these results, the selected PCA configurations are: three PCs for the ^80,84^ Kr isotopes, two PCs for ^36,40^Ar, and one PC for ^12,13^C. This optimized selection significantly reduces model complexity, easing hardware implementation on the SoC FPGA without compromising classification accuracy.

To further validate the adequacy of the selected number of PCs for each isotope pair, we employ an additional widely used FOM called “*M*”. This metric allows us to further assess the discriminative power of the random forest classifiers beyond accuracy alone. This FOM is also a well-established method in the field of signal discrimination [9,11,14] as it quantifies the separation between two overlapping distributions. It is given by

(1) $$M=\frac{\|{\vec{\mu }}_{1}-{\vec{\mu }}_{2}\|}{(\sigma_{1}+\sigma_{2})\;\cdot \;2.35}$$

where $\mu_{1}$ and $\mu_{2}$ are the means of the two distributions (representing different isotopes), and $\sigma_{1}$ and $\sigma_{2}$ are their respective full widths at half maximum. The higher M is, the better class separation is achieved:

- M>0.75, indicates satisfactory discrimination.
- M>1.0, reflects excellent class separation with minimal overlap.

Table 2 presents the FOM values for each isotope pair based on the selected number of principal components. In all cases, the FOM exceeds the 1.0 threshold, confirming that the chosen number of PCs ensures excellent isotope discrimination.

### 4.3. Hyperparameter Tuning for Random Forest

In the previous section, the selection of the number of principal components (PCs) for each isotope pair was determined using the default hyperparameters of the random forest classifier. The next step involved tuning the random forest hyperparameters to minimize computational complexity—crucial for efficient deployment on an SoC FPGA—while maintaining accuracy. The tuning process focused on key hyperparameters that directly impact both model performance and hardware resource usage: the number of decision trees (n_estimators), the maximum depth of each tree (max_depth), the number of features considered at each split (max_features), the minimum number of samples at a leaf node (min_samples_leaf), and the splitting criterion (Gini or entropy) [15].

The initial hyperparameter optimization was conducted using GridSearchCV [16,17], which exhaustively explores combinations of parameter values to identify high-performing configurations. While this method identified high-performing models, it often resulted in complex configurations that were not an optimal choice for FPGA implementation. For example, in the ^80,84^Kr isotope pair, GridSearchCV achieved 97.60% accuracy with 100 trees and a maximum depth of 80. To reduce complexity, manual tuning was then applied, guided by insights from the Grid Search results. This approach aimed to simplify the models while maintaining comparable accuracy. In the case of ^80,84^Kr, the number of trees was reduced to 15 and the maximum depth limited to 20, achieving 97.26% accuracy, a slight decrease but with significantly lower computational demands.

**Figure 5:**
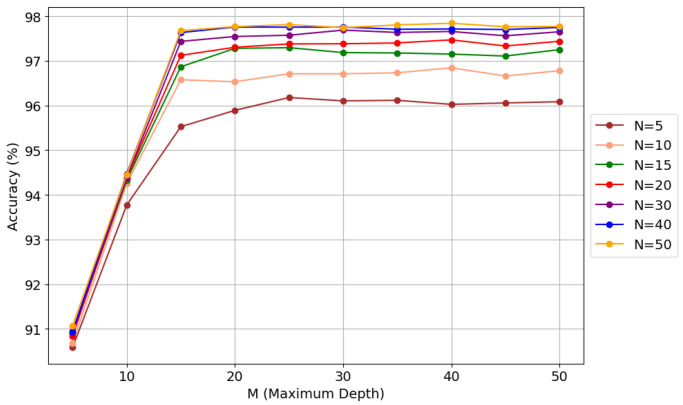
Manual hyperparameter tuning in random forest for ^36,40^Ar.

A similar process was applied to the ^36,40^Ar pair, where GridSearchCV achieved 98.00% accuracy with 100 trees and depth 80. Manual tuning simplified the model to 30 trees and depth 30, improving accuracy to 97.68% while drastically reducing complexity. Figure 5 illustrates the relationship between the number of trees (*N*) and the maximum depth (*M*) in the random forest model, along with their impact on classification accuracy. This analysis allows for evaluating the effect of hyperparameter tuning on classifier performance, facilitating a balance between accuracy and computational efficiency.

For ^12,13^C, GridSearchCV achieved 99.28% accuracy with 80 trees and depth 80, but manual refinement further optimized the model to 15 trees and depth 20, improving accuracy to 99.21%. Table 3 summarizes the results from the default settings, GridSearchCV, and manual tuning for each isotope pair, including the key hyperparameters for both tuning methods.

### 4.4. Model Compression via PCA Quantization

To further enhance computational efficiency and reduce memory usage, model compression was implemented by quantizing the PCA eigenvalues without significantly compromising classification accuracy. Quantization transforms continuous PCA eigenvalues into discrete representations, reducing the numerical precision of calculations and, consequently, computational and memory resource consumption while maintaining a minimal impact on model performance. This transformation is crucial for embedded hardware implementations, where resources are limited and storage and data processing must be optimized. Note that these elements represent the bottleneck from a computational point of view. A 4-bit quantization over the PCA eigenvalues provides an effective trade-off between classifier performance and resource efficiency. This value was chosen based on empirical evaluations [18]. The raw data was maintained at the 8-bit resolution corresponding to the resolution of the ADC used for data acquisition in order to demonstrate the viability of the proposed system with typical values presented by the acquisition systems used.

**Figure 6:**
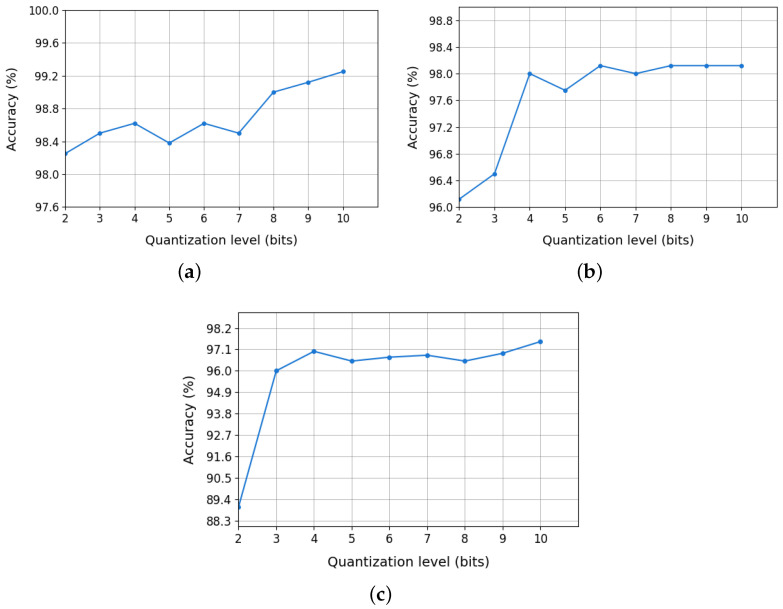
Impact of PCA eigenvalue quantization on random forest accuracy: (**a**) ^12,13^C, (**b**) ^36,40^Ar, and (**c**) ^80,84^Kr.

Finally, Figure 6 presents the accuracy of the random forest model, previously selected in Section 4.3, as a function of PCA eigenvalue quantization at different bit levels. The results indicate

- **^12,13^C:** classification accuracy remained high (98–99%) even with 4–5-bit quantization.
- **^36,40^Ar:** an optimal quantization range of 4–6 bits was identified, where accuracy degradation was minimal.
- **^80,84^Kr:** high classification accuracy was maintained with quantization down to 4 bits.

Therefore, the PCA eigenvalues will be quantized using 4 bits for all isotope pairs.

## 5. Implementation

**Figure 7:**
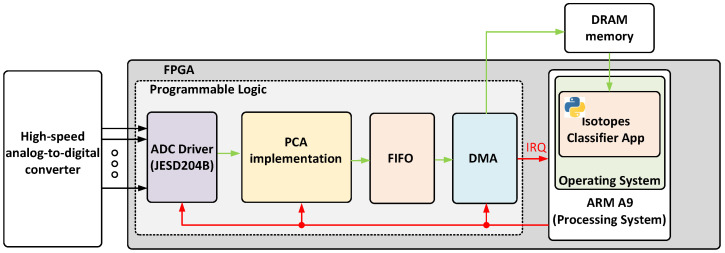
Block diagram of the proposed implementation.

This section describes both the hardware and software implementation of the proposed real-time isotope classifier. The design was developed in VHDL, using Vivado 2022.2 and deployed on a ZC706 evaluation board manufactured by AMD Xilinx [19]. This board includes a System-on-Chip (SoC) FPGA from the Zynq Z7000 family, which integrates a Dual-Core ARM Cortex A9 that runs up to 866 MHz with programmable logic that includes 350 logic cells, 19.1 Mb of block RAM, and 900 DSP slices. The CPU is responsible for running Ubuntu 20.04, while the logic fabric implements the real-time data processing pipeline. Figure 7 represents a block diagram of the proposed implementation. Each module is described in the following subsections.

### 5.1. Programmable Logic

The proposed hardware architecture is optimized for the efficient acquisition and processing of ADC-generated samples. It is composed of four main components: an ADC driver implementing a JESD204B interface, a principal component estimator (PC estimator), a memory buffer, and a DMA controller for transferring results to DRAM. It should be noted that AXI has been chosen as the communication bus protocol between the different blocks in order to facilitate its integration into the firmware developed for data acquisition systems such as [20] or [21].

**Figure 8:**
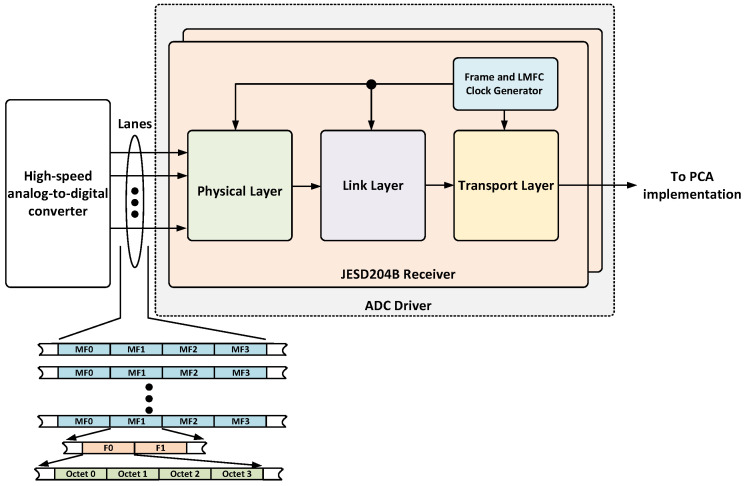
Block diagram of the ADC driver.

The ADC controller (Figure 8) is adapted from the Analog Devices JESD204B controller and manages high-speed ADCs via a JESD204B subclass 1 interface [22]. This interface ensures deterministic latency by using an external reference signal. The controller includes the physical and link layers, performing essential tasks such as clock recovery, character alignment, de-serialization, data decoding, descrambling, and redundancy verification. A supervisor module continuously monitors errors and reports them via an AXI Lite interface. During synthesis, the user can configure the physical and link layers by selecting the number of physical lanes (L), octets per frame (F), per multiframe (K), as well as the ADC resolution (N) and number of control bits used per sample (C). The selected parameters for the implementation are summarized in Table 4 based on the signal specifications presented in Section 2. The output of the link layer is delivered to the transport layer, which is in charge of handling the JESD204B deframing of the payload data. In order to simplify the integration of this component, the transport layer was modified to implement an AXI stream interface. Figure 8 shows a block diagram of the ADC driver and an example of the output, where ‘octets’ represents one data sample, ‘F’ corresponds to a frame (a group of octets for transmission), and ‘MF’ means ‘multiframe’ and corresponds to a group of multiple frames that are transmitted as a single unit.

In order to ensure that the response of one isotope pair does not affect the classification of others, the ADC controller includes a signal that activates when the ADC samples exceed a configurable threshold via a register accessible through an AXI Lite interface. When this signal is activated, the PC estimator begins calculating the different isotopes. Each one will then process 100, 200, and 300 samples for carbon, argon and krypton, respectively. When the acquired samples fall below the threshold, the trigger signal deactivates, restarting each block and preparing it for processing the next event to be classified.

**Figure 9:**
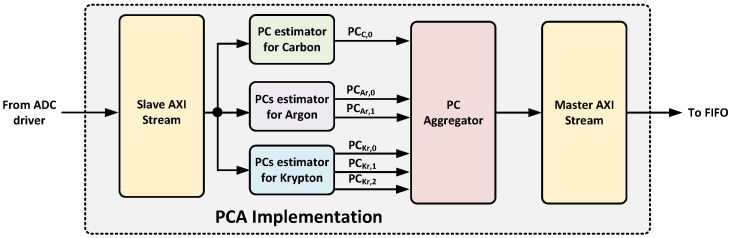
Block diagram of the PCA implementation component.

The second component, called PCA implementation, determines the principal components of the incoming data for each isotope. As shown in Figure 9, it implements a slave AXI stream interface to receive data from the ADC driver and a master AXI stream interface to deliver the principal components to the next processing stage.

**Figure 10:**
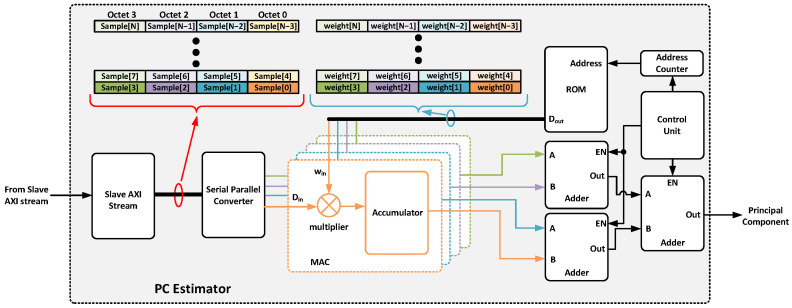
Detailed block diagram of the principal component estimator implemented.

The principal component estimator (Figure 10) leverages the FPGA’s DSP slices. Specifically, the multiply–accumulate cell (MAC) is used to compute weighted sums of the input data. The weights are stored in ROM memory that is initialized with the appropriate quantized values during synthesis. Finally, the result of the principal component estimation will be returned through a specific output port. Specifically, the block associated with isotopes ^12,13^C will present a single output port due to only one principal component being needed. For the rest of the isotopes, argon and krypton, the number of output ports will be two and three, respectively. Each time a frame is received from the ADC driver (four samples), each sample is delivered to an MAC cell to calculate the partial result of the principal component. This reduces the estimation time by a factor of F (JESD204B octets per frame). Once the total number of samples has been received, the partial results are summed and sent to the next block. Therefore, the estimation time of each principal component requires

(2) $$T_{PCA_{E}ST}=\frac{N_{FEATRURES}}{F}\;\cdot \;T_{clk,acq}+T_{sum}$$

where $N_{FEATURES}$ represents the total number of features of each isotope, $T_{clk,acq}$ is the ADC sample rate (for this application, 1 GHZ), $T_{CLK}$ is the system clock period (250 MHz), and $T_{sum}$ corresponds to the time required by the adders to generate the final result (e.g., 2 $T_{CLK}$). Therefore, the proposed architecture allows the system frequency to be reduced by a factor of F, which reduces power consumption and relaxes the timing specifications of the entire system.

In addition to the above, the PCA estimator includes a timestamp generator whose value is read once the estimation starts and is concatenated to the result of the PCs along with a field that acts as an isotope identifier. These values have been incorporated to uniquely identify each impact and simplify its subsequent processing in the user application that runs the random forest algorithm.

**Figure 11:**
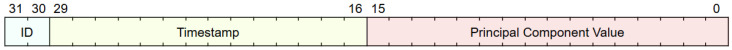
Format of the data stored by the PC aggregator in the FIFO memory.

The third key component corresponds to the PC aggregator that stores the PCA results for each isotope in FIFO memory. The data is stored as a 32-bit word whose format is shown in Figure 11. Once the FIFO memory is almost full, the DMA moves the information from the FIFO to the DRAM memory. After data transfer is completed, an interruption is triggered and handled by the ARM processor running the operating system, as described in Section 5.2. This approach reduces the load on the processor by minimizing the number of interrupts. Moreover, the PC aggregator includes some registers accessible from the user space to determine the state of execution of the system, as well as to perform actions such as restarting modules independently or disabling any of the PC estimator components.

### 5.2. Deployments of Random Forest on SoC FPGA

**Figure 12:**
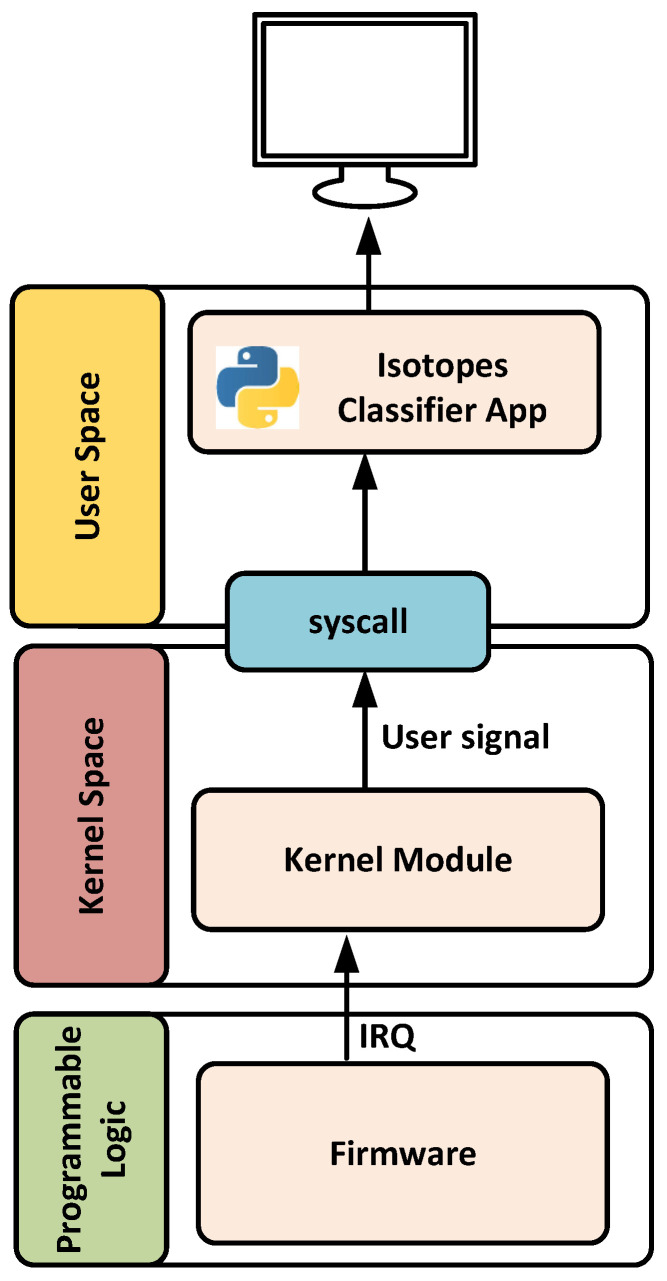
Architecture of the developed application.

This subsection describes the software application developed for isotope classification after the principal components are estimated for each hardware PC block. The application consists of two main components: a kernel module for handles interrupts and a user-space application responsible for running the classifier and transmitting data to a remote computer over Ethernet. Figure 12 illustrates the software architecture developed.

The kernel module is designed to handle interrupts generated by the DMA controller once data is successfully transferred to DRAM. Upon receiving an interrupt, the module stops the DMA, changes the destination address to a new memory location to prevent data overwriting, and then restarts the DMA operation. After this process, a user-level signal is generated to notify the user-space application that new data is available for processing and execute the classifier.

The user-space application developed in Python remains idle until it receives the signal from the kernel. Once triggered, it reads the specified DRAM memory region and transmits the information (PCs) to the cloud for isotope classification. Additionally, it supports real-time local processing of selected results. To achieve this, the application determines which isotope corresponds to the principal component, making use of the isotope identifier and timestamp (see Figure 11), and executes the associated random forest model. Depending on the isotope type, the number of memory positions accessed may vary. For example, two or three consecutive memory words are read for argon and krypton isotopes, respectively.

## 6. Performance Evaluation

The overall performance of the proposed system is assessed by two key metrics: the hardware resources utilized in the FPGA and the accuracy and latency of the classifier.

### 6.1. FPGA Resource Utilization

The proposed system was implemented on an AMD Xilinx ZC706 SoC FPGA platform. As explained in Section 5.1, the PC estimator benefits from the inherent parallelism offered by the FPGA fabric, allowing for fast and efficient computation of the principal components with minimal latency. Meanwhile, the execution of the random forest algorithm in the processor allows the classification to be performed online, in the ARM processor, or offline, sending the data obtained from the PCA execution over Ethernet for subsequent classification and storage.

Table 5 summarizes the resource utilization, in terms of lookup tables (LUTs), flip-flops (FFs), and Digital Signal Processing (DSP) cells, for each module in the hardware design.

Lastly, the resources utilized represent 1.67%, 0.94%, and 4.67% of LUTs, FFs, and DSP cells, respectively, of the ZC706 SoC FPGA platform. The minimal resources required by the proposed system enable the implementation of new discriminators for other isotopes or the simultaneous processing of multiple spectroscopy channels.

### 6.2. Latency and Inference Times

In order to evaluate the performance of the proposed system, both the latency introduced by PCA computation and the inference time of random forest classifier will be analyzed. The results provided in this section were obtained using an analog-to-digital converter model that provides samples through a JESD204B interface. The values of the samples correspond to the dataset described in Section 2.

It is important to note that the hybrid architecture proposed in Section 5 (hardware for PCA and software for random forest) allows the PC estimator component to continue to calculate the principal components of the subsequent samples and store them until the random forest classifier has completed the previous inference and can process them. Consequently, two different scenarios are considered for classifier execution. The latencies and inference times obtained for the PCA and random forest algorithms are detailed below.

Latency refers to the time between the reception of the final data frame required for an isotope and the availability of its corresponding principal component values. This process requires seven system clock cycles for final aggregation after on-the-fly computation of weighted samples. Depending on the isotope, the total latency is

- 25 clock cycles for the ^12,13^C pair.
- 50 clock cycles for the ^36,40^Ar pair.
- 75 clock cycles for the ^80,84^Kr pair.

Inference time depends on the system that runs the random forest classifier. Three deployment scenarios were considered:

1. **Scenario 1:** PCA executed in the FPGA’s programmable logic, while random forest was executed on the ARM processor.
2. **Scenario 2:** PCA executed in the FPGA, with the principal components transmitted to a remote server for classification. This remote computer will be responsible for running the random forest classifier and storing the results in a database. In our deployment, a server equipped with 2× Intel©Xeon Gold^TM^ 6430 and 128GB of RAM runs the classifier and stores the results.
3. **Scenario 3:** PCA and random forest are executed on the remote server.

Table 6 shows the average inference time per test sample for each isotope pair under both scenarios. It should be noted that the inference times correspond to the average value of 50 executions of the random forest classifier. Additionally, the transmission of information to the server located one hop away using a Gigabit Ethernet interface has been taken into account.

### 6.3. Analysis and Discussion

From the results described above, it can be seen that, once the principal components are computed, all the information needed to classify the isotope pairs is available. Consequently, the system’s maximum supported particle impact rate is determined by the acquisition time and PCA computation. Among all the considered isotope pairs, ^80,84^Kr demands the highest acquisition effort, requiring the extraction of 300 features, which translates into approximately seventy-five acquisition clock cycles, followed by seven additional clock cycles for principal component estimation. Given system clock frequencies of 250 MHz for the ADC driver and 125 MHz for the rest of the system, this setup allows for an impact rate of up to 2.8 MHz per detector, meaning it can process at edge up to 2.8 million particles per second per channel.

An important advantage of the proposed architecture is its potential scalability to multi-channel systems due to the low number of resources used. The maximum number of channels or detectors supported is limited by the number of DSP cells and/or GTX transceivers available. In our case, the ZC706 board supports up to four detectors simultaneously as the limitation is imposed by the number of GTX transceivers available.

A comparative analysis was also conducted to evaluate the advantages of the proposed system in terms of hardware efficiency and scalability. The results are presented in Table 7, which summarizes the utilization of FPGA logical resources, including lookup tables (LUTs), flip-flops (FFs), and DSP blocks, for each isotope pair considered compared to the system developed by Jiménez et al. [2]. This publication was chosen because it uses the same dataset as this study and also implements a neural-network-based isotope discriminator on an FPGA. This enables a fair and accurate comparison of the performance of our system. To the best of the authors’ knowledge, no other publications providing comparable information for this type of analysis have been identified.

The Jiménez et al. [2] implementation was specifically tailored for the ^12,13^C isotope pair and used an 8–8–2 multilayer perceptron (MLP) entirely implemented in programmable logic. While functional, this configuration required a significant amount of FPGA resources: 2304 LUTs and 1161 FFs, with no use of DSP blocks. In this approach, programmable logic handled the entire computational load, which constrained both its flexibility and scalability for more complex applications.

In contrast, the proposed system adopts a hybrid approach. This architecture combines principal component analysis (PCA) implemented in hardware with a random forest classifier executed on the ARM processor embedded within the SoC FPGA. This design achieves the same task using only thirty-four LUTs, forty-one FFs, and seven DSPs, representing a reduction of over 98% in programmable logic usage. The modularity of the system is further demonstrated by its ability to address more complex isotopic pairs, such as ^36,40^Ar and ^80,84^Kr. Even in these scenarios, the resource consumption remains low: 86 LUTs and 14 DSPs for ^36,40^Ar and 187 LUTs and 21 DSPs for ^80,84^Kr. These results highlight the adaptability of the design, enabling efficient integration into multi-channel systems without compromising performance.

Another important aspect to consider in these systems is latency, i.e., the time it takes for the system to obtain the samples and return the isotope discrimination result. The architecture presented in [2] achieved a latency of almost 1000 clock cycles to analyze a pulse and a classification accuracy of around 99%. In contrast, the system proposed in this work is capable of simultaneously discriminating the three isotope pairs with a fixed latency of seven clock cycles, which represents a significant time reduction without compromising accuracy. Table 7 and Table 8 collect the resources and inference times for each work. Note that [2] was only implemented for ^12,13^C. Thus, the resource utilization for the other isotope pairs is not available.

Finally, the accuracy of the proposed solution will be discussed. To this end, a comparison will be made with other published methodologies. This comparison will be carried out using the FOM described by Equation (1). Table 9 shows the FOM obtained by each methodology, including this work. On the one hand, the proposed approach outperforms the accuracy achieved by the methodologies proposed in [11]. On the other hand, when this work is compared to the methodologies proposed by [2,9], the FOM obtained is similar. Nevertheless, given that M>1, which ensures an excellent isotope discrimination and allows an efficient hardware implementation, the difference with the metrics obtained by [2,9] for the isotope ^12,13^C is not relevant.

## 7. Conclusions

This paper presents a hardware–software co-design system for isotope discrimination based on SoC FPGAs. For this purpose, the principal component analysis algorithm—responsible for obtaining the most relevant features of the acquired samples—and the random forest classifier are used. The first algorithm, PCA, is efficiently accelerated in the programmable logic of the FPGA, while the classifier runs on the ARM processor that is included in the selected SoC FPGA (AMD Xilinx ZC706). The proposed solution takes advantage of the parallelism and low power consumption of the logic fabric, as well as the computational power and versatility of the ARM processor.

To achieve efficient implementation, we first estimated the number of principal components required for each pair of isotopes. Next, we optimized the classifier hyperparameters to maximize classification accuracy. After this procedure, a hardware accelerator was developed to estimate the principal components for each isotope pair. Finally, a Python-based application was developed that loads the trained models and data, processes and classifies a given isotope, and prints the prediction in a readable label (^12^C, ^13^C, ^36^Ar, ^40^Ar, ^80^Kr, and ^84^Kr). This function enables effective isotope classification, essential for applications that require accurate and fast identification of different isotopes.

The main advantages of the proposed system include a significant reduction in running time for PCA computation compared to its implementation on a microprocessor, as well as an overall improvement in resource utilization. By offloading the most computationally intensive tasks to dedicated hardware, the system achieves better performance while maintaining flexibility for the subsequent processing stages.

The implementation of the solution on the ZC706 platform has allowed its performance to be validated, confirming that the data transfer between the programmable logic and the processing system has minimal latency and makes efficient use of the hardware resources. In addition, the ability to classify isotopes offline means that detection capacity is not limited by inference time. It is also important to highlight that, due to the reduced number of resources used, the system can integrate more discriminators or increase the number of spectroscopy channels that it can handle simultaneously.

Based on the results, the solution presented improves the accuracy and resources used by other systems proposed in the literature. However, from the point of view of latency, due to the limited computing capacity of the ARM processor, isotope discrimination is negatively impacted.

An additional scenario was evaluated in which both PCA and random forest execution were carried out entirely on the remote server. This configuration highlights the advantages of the proposed system as a computer with higher processing capabilities that requires more time to compute the principal components, although at the cost of increased energy consumption.

Future work will explore adaptive strategies in which the optimal number of PCs is dynamically selected based on the characteristics of the real-time signal. This will enable the system to adapt to changing acquisition conditions or new isotope signatures, enhancing its generalizability without incurring substantial additional computational costs. The proposed solution can adapt to possible changes by reprogramming the logic fabric of the SoC FPGA in real time. This is made possible by the parameterization of the HDL code that has been developed.

In summary, the presented system offers an effective solution for real-time or offline isotope discrimination by combining the advantages of hardware acceleration with the versatility of software classification.

## Figures and Tables

**Table 1:** Dataset for each isotope.

Isotope	Observations	Features
**^80,84^Kr**	1100	300
**^36,40^Ar**	2000	200
**^12,14^C**	2000	100

**Table 2:** FOM for each isotope pair.

Isotope	Number of PCs	FOM
^80,84^Kr	3-PCs	1.10
^36,40^Ar	2-PCs	1.46
^12,13^C	1-PC	2.50

**Table 3:** Summary of model performance.

Isotope	Method	Accuracy	N_Estimators_	Max. Depth
^80,84^Kr	Default	97.95	100	None
	GridSearchCV	97.60	100	80
	Manual tuning	97.26	15	20
^36,40^Ar	Default	97.75	100	None
	GridSearchCV	98.00	100	80
	Manual tuning	97.68	30	30
^12,13^C	Default	99.50	100	None
	GridSearchCV	99.28	80	80
	Manual tuning	99.21	15	20

**Table 4:** JESD204B configuration parameters used during synthesis.

Parameter	Value
Number of lanes	1
Number of octets per frame	4
Number of frames per multiframe	1
ADC resolution [bits]	8
Number of control bits	0

**Table 5:** Utilization of SoC FPGA for the proposed system.

Isotope	LUT	FF	DSP
ADC Driver	2285	2565	0
PC estimator for Carbon	34	41	7
PC estimator for Argon	86	85	14
PC estimator for Krypton	187	137	21
PC Aggregator	404	714	0
FIFO	53	101	0
DMA	601	1249	0
Total	3650	4089	42

**Table 6:** Inference time obtained for each scenario.

Inference Time [ms]			
**Isotope**	**Scenario 1**	**Scenario 2**	**Scenario 3**
^12,13^C	17.2198	0.8712	0.8807
^36,40^Ar	31.4734	1.6158	1.6308
^80,84^Kr	17.0567	0.9047	0.9118

**Table 7:** Comparison of FPGA resource utilization between the neural network approach by Jiménez et al. [2] and the proposed PCA + random forest system for each isotope pair.

Resource	Jiménez et al. [2]	This Work		
	**^12,13^C**	**^12,13^C**	**^36,40^Ar**	**^80,84^Kr**
**LUTs**	2304	34	86	187
**FFs**	1161	41	85	137
**DSPs**	0	7	14	21

**Table 8:** Comparison of inference times.

Inference Time	Jiménez et al. [2]	This Work					
	**^12,13^C**	**^12,13^C**		**^36,40^Ar**		**^80,84^Kr**	
	**ANN [μs]**	**PCA [μs]**	**Random** **Forest [μs]**	**PCA [μs]**	**Random** **Forest [μs]**	**PCA [μs]**	**Random** **Forest [μs]**
Scenario 1	6.99	0.156	17,219.8	0.256	31,473.4	0.356	17,056.7
Scenario 2	Not Applied	0.156	869.6	0.256	1614.2	0.356	903.1

**Table 9:** Factors of merit among different methods.

Methods	Units	Reference	M per Isotope Pair		
			**^12,13^C**	**^36,40^Ar**	**^80,84^Kr**
Amplitude	[mA]	[11]	1.42	0.81	0.54
Risetime	[ns]	[11]	0.62	0.36	0.26
Decay time	[ns]	[11]	0.81	0.48	0.007
Slope	[mA/ns]	[11]	1.35	0.73	0.11
$m^{2}$	[ns]	[11]	0.91	0.64	≈0
f[i] current signal	-	[11]	1.15	0.84	0.50
data[i] current signal	-	[11]	1.53	0.96	1.04
Standard ANN	-	[1]	1.71	0.76	0.98
Differential ANN	-	[1]	4.48	0.90	2.95
Linear SVM	-	[9]	3.84	0.66	1.78
Cubic SVM	-	[9]	7.50	1.04	2.04
Random Forest	-	This work	2.50	1.46	1.10

## Data Availability

Data available on request.

## Abbreviations

The following abbreviations are used in this manuscript:

ADC	Analog-to-Digital Converter
DPSA	Digital Pulse-Shape Analysis
FOM	Figure of Merit
FPGA	Field-Programmable Gate Array
FWHM	Full Width at Half Maximum
NTD	Neutron Transmutation Doping
PC	Principal Component
PCA	Principal Component Analysis
SoC	System-on-Chip
SQL	Structured Query Language
